# Treatment Outcomes of Tuberculosis and Associated Factors Among Tuberculosis and Human Immunodeficiency Virus Co-infected Patients in Public Health Facilities in Jigjiga, Somali Region, Ethiopia

**DOI:** 10.7759/cureus.56092

**Published:** 2024-03-13

**Authors:** Abdilahi Omer Abdilahi, Ayan Abdishukri Ahmed, Mohamed Omar Osman

**Affiliations:** 1 Public Health, Jigjiga University, Jigjiga, ETH; 2 Maternal and Child Health, Jigjiga University, Jigjiga, ETH

**Keywords:** somali region, jigjiga, risk factors, tb treatment outcome, s: tb-hiv co-infected

## Abstract

Background: Worldwide, tuberculosis (TB) is a serious public health issue, especially in low-income countries, including Ethiopia. For those who are HIV-positive, TB poses a major risk to their health. The development of chemotherapy and the effectiveness of treatment have resulted in notable increases in patient survival. The evaluation of TB treatment outcomes is an essential metric for determining the success of TB and HIV co-morbidity control strategies.

Purpose: This study aims to identify TB treatment outcomes and associated factors among TB/HIV co-infected patients in public health facilities in Jigjiga, Somali Region, Ethiopia, in 2021.

Patients and methods: A hospital-based cross-sectional study design was done on three facilities (Karamara, Hasan Yabare Referral Hospital, and Jigjiga Health Center) with a total of 194 study participants. Data were extracted using a checklist, entered into EpiData version 3 (The EpiData Association, Odense, Denmark), and analyzed using SPSS Statistics version 20 (IBM Corp. Released 2011. IBM SPSS Statistics for Windows, Version 20.0. Armonk, NY: IBM Corp.) for descriptive and inferential analysis of the study objectives. Variables in the bivariate logistic regression analysis with p-values less than 0.25 were entered into a multivariate logistic regression to identify the independent factors of TB treatment outcome. Associations were computed using an adjusted odds ratio with a 95% CI. P-values less than 0.05 were finally considered statistically significant.

Results: The following TB treatment outcomes were observed among all TB/HIV co-infected patients enrolled in this study: 126 (67.4%) completed treatment, three (1.8%) died, 42 (22.5%) were cured, and 16 (8.6%) were transferred out; 168 (89.8%) had a successful treatment outcome.

Category of the patient (AOR = 0.194, 95% CI: 0.041, 0.923), sex of the patient (AOR = 1.490, 95% CI: 1.449, 4.951), and cotrimoxazole preventive therapy (CPT) initiation (AOR = 0.073, 95% CI: 0.021, 0.254) were found to be significant predictors for successful TB treatment outcome at a p-value less than 0.05 with a 95% CI.

Conclusion: Overall, 89.8% of TB treatments were successful among TB/HIV co-infected patients. This study has found sex, socioeconomic status, and CPT initiation were significant factors for a successful TB treatment outcome. Based on these findings, governmental and non-governmental organizations should facilitate the implementation and enforce the availability of all TB/HIV co-infected patients.

## Introduction

Tuberculosis (TB) is an infectious, airborne disease that has become a major global health problem. Each year, there are around 9 million new cases of TB and 1.5 million deaths, of which 360,000 are HIV-positive patients [1]. It is a disease caused by a bacterium species called *Mycobacterium tuberculosis*. It spreads via respiratory transmission [2]. HIV/AIDS is a disease of the human immune system caused by infection with HIV [3].

Globally, in 2014, there were an estimated 1.2 million new HIV-positive TB cases, and almost three-quarters of these cases were in the African region [4]. The difficulties in managing TB/HIV co-infected individuals include pill burden, increased adverse effects, drug-to-drug interactions, and immune reconstitution inflammatory syndrome. HIV/AIDS has a number of impacts on the prevention and control of TB [5]. TB is the second-leading cause of death globally due to a single infectious agent [6]. However, it is a leading preventable cause of death among people living with HIV and vice versa [7].

National TB programs face numerous challenges as a result of the expanding HIV epidemic, including an increase in HIV infection among patients with TB and the emergence of new TB cases among HIV-positive individuals. The efficacy of the National Tuberculosis Program and the performance of the health system are being jeopardized as a result of rising TB incidence, case mortality, treatment discontinuation, and difficulties in providing both diseases with comprehensive care [8].

A study from several nations has shown that anti-TB therapy regimens have a treatment success rate of between 30% and 83%. The treatment outcomes are significantly correlated with clinical variables such as HIV/AIDS co-infection, TB category, and late antiretroviral therapy (ART) beginning, as well as socio-demographic characteristics such as age, sex, and marital status [9-12].

ART can reduce the risk of death from TB from 64% to 95%. ART should be begun two to eight weeks after beginning anti-TB medication because of this advantage. HIV-positive TB patients have a longer infectious period, which makes TB therapy more difficult for them [13].

The WHO Global TB Report of 2018 estimated that 10.0 million new cases of TB occurred in 2017, of which over 82% of TB deaths occurred in low- and middle-income countries. In 2017, there were an estimated 0.9 million new cases of TB among HIV/AIDS patients, 72% of whom lived in Africa. TB was a leading killer of people living with HIV/AIDS in 2017, with 0.3 million TB-related deaths [14].

In low-income nations, co-infection with HIV and TB is a serious public health risk. For example, up to 30% of TB cases in Africa are HIV-positive. Moreover, in Ethiopia's neighbor, Sub-Saharan Africa, up to 80% of patients with active TB also have HIV [15].

Ethiopia is one of the countries particularly affected by TB/HIV co-infection; it is ranked seventh out of 22 countries with high rates of TB burden worldwide. Evaluating the potential causes of poor treatment results in people with co-infections with HIV and TB is an ongoing need. This is particularly critical in developing nations such as Ethiopia, where low literacy, unstable economies, and restricted access to healthcare facilities all raise the risk of unfavorable consequences [14].

According to a study conducted, the prevalence of TB among people living with HIV was reported to be 14.5% in the Somali Region [16]. Socioeconomic development is further impeded by TB, which affects 75% of those in the 15-54 age range that is considered economically productive. The TB crisis was exacerbated by the HIV epidemic; in contrast to HIV-negative individuals, who have a 10% lifetime risk of developing TB disease, 50% to 60% of HIV-infected individuals are expected to develop TB disease over their lifetime [17].

Treatment outcomes serve as a proxy for the quality of TB treatment provided by the health care system. There is little data about the treatment outcome of TB among TB/HIV co-morbidity patients in the Somali Region in general and Jigjiga in particular. The study provides baseline information on the topic in the study area and helps to undertake further study on a large scale. Therefore, this study aims to identify TB treatment outcomes and associated factors among TB/HIV co-infected patients in the public health facilities in Jigjiga, Somali Region, Ethiopia, in 2021.

## Materials and methods

Study area and period

This study was conducted among public health facilities in Jigjiga, Somali Region, Ethiopia. Jigjiga is the capital of the Somali Regional State, which is located in eastern Ethiopia. According to Central Statistical Agency predictions for the 2015 Ethiopian fiscal year, Jigjiga has a total population of 426,122, of which 85,650 are expected to be in the reproductive age range (15-49 years). The city has thirty sub-districts, the smallest administrative units being urban (20) and rural (10). Ninety-seven percent of the population is in Somalia, and 98% of them are Muslims [17]. In the study area, there are three health facilities, one regional hospital, and one referral hospital. Jigjiga Health Center, Hassan Yabare Referral Hospital, and Karamara were the sites of the study. Comprehensive medical records of individuals co-infected with HIV and TB were examined to extract patient socio-demographic information, medication history, type of TB, and treatment results. This study period was between February 2021 and August 2021.

Study design

An institutional-based retrospective study was conducted by reviewing 60 months (January 2015 to January 2020) of medical records of TB/HIV co-infected patients to identify TB treatment outcomes and associated factors among TB/HIV co-infected patients at public health facilities in Jigjiga, Somali Region, Ethiopia.

Source and study population

All TB/HIV co-infected patients’ records in Jigjiga public health facilities who attended anti-TB therapy were the source population in this study period. TB patients who had started a course of anti-TB therapy and had complete patient records within Jigjiga public health facilities from January 2015 to January 2020 were the study population.

Inclusion and exclusion criteria

All of the patients with complete medical records who registered in these hospitals between January 2015 and January 2020 and who were co-infected with TB and HIV were included. The medical records of patients co-infected with TB and HIV between January 2015 and January 2020 who were transferred out and the records of patients whose variables of interest were missing were excluded from the study.

Sample size determination

The sample size was determined by using a single proportion population formula with a 95% confidence level, a 5% margin of error, and an 86.8% proportion of successful treatment outcomes among TB/HIV co-infected patients in Harar, Ethiopia [8]. Hence, the minimum sample size was calculated as follows: by considering a 10% non-respondent rate for the final sample size, n = 176 + 17.6 = 193.6 = 194.

Sampling technique

A sampling frame consisting of all TB- and HIV-positive patients registered for anti-TB medication between January 2015 and January 2020 was used to choose 194 study participants. A basic random sampling technique was employed to choose specific patient records from the corresponding public health facilities. The first three public health institutions (Jigjiga Health Center, Hassan Yabare Referral Hospital, and Karamara General Hospital) were included purposefully. Every case of TB that was documented at a public health facility was considered. Every patient whose profile was co-infected with HIV and TB was then evaluated in each hospital to make sure the inclusion requirements were fulfilled. Finally, research participants who met the inclusion requirements were selected from the TB registration book until the target sample size was reached.

Data collection techniques and tools

The data was collected using a semi-structured checklist-style data extraction format. All pertinent socio-demographic information, clinical variables (sputum smear, TB type, patient type, HIV status, medication regimen, and treatment outcomes), laboratory results, and follow-up data were included in the standardized checklist that was utilized. TB treatment registry, monthly cohort form, and follow-up form) of the patients were reviewed in order to obtain the data. A data extraction method was created after taking the variables under study into consideration. Each hospital's TB clinic head nurse or health officer, together with data collectors, attended a half-day instruction on capturing the precise information from the follow-up card, excluding partial cards, and inclusion and exclusion criteria. Four BSc nurses who had training in comprehensive TB care and experience in data collection were used to collect the data.

Study variables

The dependent variable is the outcome of TB treatment (successful treatment vs. unsuccessful treatment). The independent variables are socio-demographic characteristics (sex, age, place of residence, pre-treatment weight, year of treatment, marital status, and educational level) and clinically related factors (TB case, duration on ART, type of TB, types of opportunistic infections, cotrimoxazole preventive therapy (CPT) initiated, WHO staging, baseline CD4 counts, and category of patient).

Data quality control

The following steps were taken to guarantee the quality of the data: eight TB/HIV co-infected patient medical records at particular public health facilities were used to pretest the extraction format, and data collectors received two days of training. The principal investigators also oversaw the entire data collection process on a daily basis and verified that checklists were consistently filled out.

Operational definitions

Unsuccessful treatment outcomes were defined as the sum of failure, death, and transfer out, while successful treatment success was defined as the sum of being cured and having treatment completed [14].

Data processing and analysis

The data were entered into EpiData version 3 (The EpiData Association, Odense, Denmark) and analyzed using SPSS Statistics version 20.0 (IBM Corp. Released 2011. IBM SPSS Statistics for Windows, Version 20.0. Armonk, NY: IBM Corp.). Descriptive statistics, such as frequency, percentage, mean, and standard deviation, were used to summarize descriptive characteristics. The study employed bivariate and multivariate logistic regression methods to examine the relationship between the independent and dependent variables. A multivariate logistic regression model was constructed using the variables that displayed a correlation in the bivariate analysis with p<0.25. The essential presumptions for the use of multivariate logistic regression and multi-colinearity were evaluated using Hosmer and Lemeshow's goodness of fit test. A multivariate logistic regression model was fitted to determine independent predictors of treatment outcomes. A p-value less than 0.05 was declared significant.

Ethical consideration

Ethical clearance for this study was obtained from the College of Medicine and Health Science and the Jigjiga University Research and Ethical Committee (approval number: 0060/2021). A letter of cooperation was obtained from the school of graduate studies for the selected health facilities. All data obtained in the course of the study were kept confidential and used solely for the purpose of the study.

## Results

Socio-demographic characteristics of study participants

From the 194 patients in the computed sample size, a total of 187 TB/HIV co-infected patients on TB treatment who met the inclusion criteria from the three health institutions were included, which makes the response rate 96.4%. The mean (±SD) age of participants was 34.91 (±11.68) years, with 88 (47.1%) of the participants being in the 31- to 45-year-old age group. Of the total participants, 101 (56%) and 86 (44%) were males and females, respectively, and 162 (86.6%) of the co-infections occurred among urban residents. Pre-treatment weights have a mean and standard deviation of 47.86 (9.42) kg (Table 1).

**Table 1:** Socio-demographic characteristics of TB/HIV co-infected patients at health facilities in Jigjiga, Somali Region, Ethiopia, 2021 TB: tuberculosis, HIV: human immunodeficiency virus

Variables	Categories	Frequency	Percentage
TB clinic	Karamara General Hospital	75	40.1
	Hasan Yabare Referral Hospital	42	22.5
	Jigjiga Health Center	70	37.4
Age	1-15 years	6	3.2
	16-30 years	69	36.9
	31-45 years	88	47.1
	>45 years	24	12.8
Sex	Male	101	54.0
	Female	86	46.0
Marital status	Married	105	56.1
	Unmarried	82	43.9
Place of residence	Urban	162	86.6
	Rural	25	13.4
Educational level	No education	99	52.9
	Educated	88	47.1
Year of TB treatment initiated	2008EC	49	26.2
	2009EC	32	17.1
	2010EC	28	15.0
	2011EC	46	24.6
	2012EC	32	17.1
Pretreatment weight	<35 kg	9	4.8
	36-45 kg	80	42.8
	46-55 kg	59	31.6
	>56 kg	39	20.9

Clinical characteristics of the TB/HIV co-infected patients

One hundred seventy-two (92.0%) of the total number of co-infected patients were classified as new cases. Regarding the type of TB, 99 (52.9%) were diagnosed with pulmonary TB, of which 80 (42.8%) were smear-positive patients who initiated ART, while 174 (93%) initiated ART, and 164 (87.7%) started CPT. The majority of co-infected patients in this study were in WHO stage III, numbering 146 (78.1%). Concerning baseline CD4 count, 123 (65.8%) fell between 201 and 499 cells/µL (Table 2).

**Table 2:** Clinical characteristics of TB/HIV co-infected patients at health facilities in Jigjiga, Somali Region, Ethiopia, 2021 ART: antiretroviral therapy, CPT: cotrimoxazole preventive therapy, TB: tuberculosis, HIV: human immunodeficiency virus, WHO: World Health Organization, STI: sexually transmitted infection, PTB: pulmonary tuberculosis

Variables	Categories	Frequency	Percentage
Type of patient	New	172	92.0
	Retreatment	15	8.0
ART initiated	Yes	174	93.0
	No	13	7.0
CPT	Yes	164	87.7
	No	23	12.3
Types of TB	PTB	99	52.9
	Extra PTB	88	47.1
Smear result	Smear positive	80	42.8
	Smear negative	107	57.2
WHO stage at TB	Stage I	2	1.1
	Stage II	9	4.8
	Stage III	146	78.1
	Stage IV	30	16.0
Functional status	Working	165	88.2
	Bed-ridden	22	11.8
Types of opportunistic infection	Pneumonia	69	36.9
	Candidiasis	40	21.4
	STI	10	5.3
	Intestinal parasites	13	7.0
	Urinary tract infections	14	7.5
	Others	41	21.9
Baseline CD4 count	Less than 200 cells/µL	26	13.9
	201-499 cells/µL	123	65.8
	Greater than 500 cells/µL	38	20.3
Duration on ART	Less than 2 years	42	22.5
	More than 2 years	145	77.5

TB treatment outcome of TB/HIV co-infected patients

Among all TB patients enrolled in this study, 126 (67.4%) completed their treatment, three (1.6%) died, 42 (22.5%) were cured, and 16 (8.6%) transferred out. Of the 187 patients identified for treatment outcome, 168 (89.8%) showed a successful treatment outcome (Table 3).

**Table 3:** TB treatment outcome among TB/HIV co-infected patients at health facilities in Jigjiga, Somali Region, Ethiopia, 2021 (n=187) TB: tuberculosis, HIV: human immunodeficiency virus

Variable	Category	Frequency	Percentage
TB treatment outcome	Cured	42	22.5
	Treatment completed	126	67.4
	Death	3	1.6
	Transfer out	16	8.6
Overall outcomes	Successful outcome	168	89.8
	Unsuccessful outcome	19	10.2

Multivariate analysis

In this study, a multivariate analysis was conducted to see the effect of combined variables on the outcome variables (successful TB treatment outcome), which were significant in the bivariate analysis (p<0.25). Thus, at a p-value less than 0.25, patient categories, type of TB, CPT initiation and WHO staging, sex, educational level, and pre-treatment weight were found to be significant factors for successful TB treatment outcomes. A multivariate analysis result indicates that variables including category of patient, sex of the patient, and CPT initiated were found to be significant factors for successful TB treatment outcome at a p-value less than 0.05.

The probability of a successful TB treatment outcome was 80.6% less likely in new TB treatment patients than in re-treatment (AOR = 0.194, 95% CI: 0.041, 0.923). However, being male, the probability of having a successful TB treatment outcome was 1.49 times higher than being female (AOR = 1.490, 95% CI: 1.449, 4.951). TB patients who started CPT during their TB diagnosis had a 7.3% higher chance of a successful TB treatment outcome (AOR = 0.073, 95% CI: 0.021, 0.254) (Table 4).

**Table 4:** Multivariate analysis of TB treatment outcome with socio-demographic and clinical characteristics of TB/HIV co-infected patients at health facilities in Jigjiga, Somali Region, Ethiopia, 2021 *significant variables, TB: tuberculosis, CPT: cotrimoxazole preventive therapy, WHO: World Health Organization, HIV: human immunodeficiency virus, COR: crude odds ratio, AOR: adjusted odds ratio, CI: confidence interval

Variables	Categories	Outcomes		COR (95.0% CI)	AOR (95.0% CI)	P-value
		Successful outcome	Unsuccessful outcome			
Category of patient	New	157 (91.3%)	15 (8.7%)	0.26 (0.074-0.93)	0.194 (0.041-0.923)	0.039*
	Retreatment	11 (73.3%)	4 (26.7%)	1	1	
Sex	Male	88 (87.1%)	13 (12.9%)	1.97 (0.712-5.43)	1.490 (1.449-4.951)	0.015*
	Female	80 (93.0%)	6 (7.0%)	1	1	
CPT initiated	Yes	154 (93.9%)	10 (6.1%)	0.101 (0.035-0.29)	0.073 (0.021-0.254)	0.000*
	No	14 (60.9%)	9 (39.1%)	1	1	
WHO staging	Stage I	1 (50.0%)	1 (50.0%)	6.5 (0.34-126)	26.347 (0.977-7.431)	0.052
	Stage II	8 (88.9%)	1 (11.1%)	0.81 (0.08-8.35)	5.656 (0.370-86.577)	0.213
	Stage III	133 (91.1%)	13 (8.9%)	0.64 (0.19-2.10)	1.906 (0.375-9.688)	0.437
	Stage IV	26 (86.7%)	4 (13.3%)	1	1	
Educational level	No education	93 (93.9%)	6 (6.1%)	0.37 (0.14-1.03)	0.420 (0.0125-1.408)	0.160
	Educated	75 (85.2%)	13 (14.8%)	1	1	
Pretreatment weight	Less than or equal to 40 kg	29 (96.7%)	1 (3.3%)	0.266 (0.034-2.075)	1.786 (0.609-5.236)	0.291
	More than 40 kg	139 (88.5%	18 (11.5%)	1	1	

## Discussion

Among all TB patients included in this study, 126 (67.4%) completed their treatment, three (1.6%) died, 42 (22.5%) were cured, and 16 (8.6%) were transferred out. From the 187 patients evaluated for treatment outcome, the overall TB treatment success among TB/HIV co-infected patients was 168 (89.8%). This finding was similar to the study conducted in Ahmedabad (84.2%) [18], South Africa (82.2%) [19], and Addis Ababa, Ethiopia (88.2%) [20]. On the other hand, the TB treatment success rate of our study is higher than several studies conducted in Gondar (22.6%) [21], Malaysia (53.4%) [22], and South West Ethiopia (30.3%) [23]. This difference may be due to the sample size and socio-economic characteristics of the study participants.

However, our finding was lower than that of the India study, which reported a treatment success rate of 93.1% [24]. This observed discrepancy may be the result of variations in the level of care provided in the TB/HIV clinic, including appropriate follow-up by the doctor, health education, and counseling. A variation in sample size could be the cause of another probable explanation.

In the multivariate analysis, category of the patient (AOR = 0.194, 95% CI: 0.041, 0.923), sex of the patient (AOR = 1.490, 95% CI: 1.449, 4.951), and CPT initiated (AOR = 0.073, 95% CI: 0.021, 0.254) were found to be significant factors for successful TB treatment outcome at a p-value less than 0.05. This study found that for TB patients who received new TB treatment, the likelihood of a successful TB treatment outcome was 80.6% less likely than for those who received re-treatment (AOR = 0.194, 95% CI: 0.041, 0.923). This finding was in line with a study conducted in Harar [8] and Cameron [25]. This demonstrates that a TB patient who was declared cured or completed could return to the health service and be found to be smear positive in the sputum, which is classified as a relapse, making treatment difficult because the patient had a history of compromised immunity and treatment failure compared to these new patients.

This study showed that the sex of the patient (AOR = 1.49, 95% CI: 1.449, 4.951) was found to be a significant factor for successful TB treatment outcomes. The probability of having a successful TB treatment outcome of being male was 1.49 times higher than being female. The finding was supported by a study that was conducted in Ethiopia and Cameron [25]. This could be because females have different maternal-related complications and are less immune-competent than males, which reduces successful treatment outcomes [26].

In this study, TB patients who began CPT during their TB diagnosis had a 92.7% less chance of a successful TB treatment outcome than those who did not (AOR = 0.073, 95% CI: 0.021, 0.254). This finding contradicts studies in Ethiopia and India [27,28], which indicated that a combination of cotrimoxazole and TB drugs increases the success of treatment because it is probable that CPT reduces mortality from other opportunistic infections, including TB. The variance may be due to patients having a history of cotrimoxazole hypersensitivity or drug resistance.

Limitations of the study

The limitation was that the data collected were retrospective and used secondary data. There were issues such as missing and/or inaccurate information on some factors like economic variables (e.g., employment, occupation, income) and individual patient factors (e.g., alcohol use/abuse and smoking) that could have influenced TB treatment success.

## Conclusions

From the patients included in this study, a considerable rate of unsuccessful treatment outcomes was identified. The category of the patient, sex of the patient, and CPT initiating factors were found to be significant factors for a successful TB treatment outcome. Based on these results, the study suggests that local managers, governmental and non-governmental organizations, and other stakeholders involved in the management of TB and HIV should make it easier for all patients who are co-infected with HIV and TB to have access to cotrimoxazole, and they should pay particular attention to patients who are re-treating HIV. Additional prospective research is needed to investigate the effects of TB treatment and related variables in TB patients who also have HIV.
